# Vinegar: A potential source of healthy and functional food with special reference to sugarcane vinegar

**DOI:** 10.3389/fnut.2023.1145862

**Published:** 2023-03-16

**Authors:** Gan-Lin Chen, Feng-Jin Zheng, Bo Lin, Yu-Xia Yang, Xiao-Chun Fang, Krishan K. Verma, Li-Fang Yang

**Affiliations:** ^1^School of Chemistry and Chemical Engineering, Guangxi Minzu University, Nanning, Guangxi, China; ^2^Institute of Agro-Products Processing Science and Technology, Guangxi Academy of Agricultural Sciences, Nanning, Guangxi, China; ^3^Guangxi Key Laboratory of Fruits and Vegetables Storage-Processing Technology, Nanning, China; ^4^Key Laboratory of Sugarcane Biotechnology and Genetic Improvement (Guangxi), Ministry of Agriculture and Rural Affairs, Nanning, Guangxi, China; ^5^Guangxi Key Laboratory of Sugarcane Genetic Improvement, Nanning, Guangxi, China; ^6^Sugarcane Research Institute, Guangxi Academy of Agricultural Sciences, Nanning, Guangxi, China

**Keywords:** fatty acid, fermentation processes, health benefits, VOCs, phytochemistry, production approaches, *Saccharum* spp., vinegar

## Abstract

Vinegar is one of the most widely used acidic condiments. Recently, rapid advances have been made in the area of vinegar research. Different types of traditional vinegar are available around the globe and have many applications. Vinegar can be made either naturally, through alcoholic and then acetic acid fermentation, or artificially, in laboratories. Vinegar is the product of acetic acid fermentation of dilute alcoholic solutions, manufactured by a two-step process. The first step is the production of ethanol from a carbohydrate source such as glucose, which is carried out by yeasts. The second step is the oxidation of ethanol to acetic acid, which is carried out by acetic acid bacteria. Acetic acid bacteria are not only producers of certain foods and drinks, such as vinegar, but they can also spoil other products such as wine, beer, soft drinks, and fruits. Various renewable substrates are used for the efficient biological production of acetic acid, including agro and food, dairy, and kitchen wastes. Numerous reports on the health advantages associated with vinegar ingredients have been presented. Fresh sugarcane juice was fermented with wine yeast and LB acetate bacteria to develop a high-quality original sugarcane vinegar beverage. To facilitate the current study, the bibliometric analysis method was adopted to visualize the knowledge map of vinegar research based on literature data. The present review article will help scientists discern the dynamic era of vinegar research and highlight areas for future research.

## Introduction

Rapid development in the food supply chain has led to an increased interest in quality in the food sector. Human health and food safety have become essential in the last few decades. Health problems are highly related to diet and nutritional habits. The connection between nutrition and the development of various health problems is even more noticeable when close attention is given to every age group. As regards the chemical composition of foods, a large number of bioactive compounds present in plants, fruits, vegetables, dairy products, meat, and fish are currently known. Bioactive compounds from food play an important role in disease prevention (1, 2). Vinegar has antibacterial properties, antioxidant activity, anti-diabetic and anti-tumor effects, and the ability to prevent cardiovascular disorders. Additionally, due to its medicinal properties, it has long been applied in traditional ancient medicine (3, 4). Every nation or location in the world has varieties of vinegar with distinctive aromas and flavors derived from the raw ingredients, microorganisms, and vinegar manufacturing processes (3, 5, 6). In addition to being used as a flavoring agent, vinegar is a functional food and beverage since it contains some healthy ingredients, particularly in the older varieties (7, 8).

Globalization and the rapid expansion of food production have resulted in new consumer expectations regarding food and balanced diets. However, due to the significant increase in life expectancy, there is an urgent need for specific foods that meet all nutritional requirements and help us maintain a healthy diet, which is essential for maintaining human health (9–11). Therefore, food industries must keep up with consumers' interests and needs while developing novel products. Additionally, health experts, food technologists, biologists, healthcare companies, and consumers tend to highlight a great deal of interest in disease prevention. Functional foods are classified as nutritious foods, medicinal foods, regulatory foods, fortified foods, nutraceuticals, and pharmacological foods. Functional foods contain nutrients that have the potential to improve human life or reduce the risk of certain abnormalities (11–13).

Fruit is a well-known source of nutrients with functional qualities. Fruit contains flavors, colors, and aromas in addition to phytochemicals with antioxidant activities. Due to their capacity to scavenge and suppress free radicals formed during the oxidative metabolism that have detrimental effects on human health, antioxidants found in fruits have already been linked to nutritional advantages (14, 15). As a result, there is great interest in developing approaches for delivering these nutrients, and vinegar represents a promising possibility for establishing an improved functional food. In addition to being used as a food condiment, vinegar is a key component in the formulation of several drinks. Consequently, it was estimated that the market for products related to vinegar and the demand for genuine, high-quality fruit vinegar products will increase (2, 16). Fruit vinegar designation is also valid for products that mix juice with vinegar. However, establishing the types of vinegar made from fruits is crucial to provide a final product with natural fruit properties, given consumers' interest in high-quality and good food products (16).

Vinegar is an acidic condiment produced from various raw materials, including grains, fruits, and vegetables, and is manufactured worldwide. Cider vinegar and regular vinegar are the two varieties. Cider vinegar is made from fruit juices. It is a highly advantageous beverage, as it helps to promote various types of beneficial effects to consumers. Additionally, cider vinegar has been reported to have the potential to balance pH levels in the body if taken regularly. Regular vinegar is manufactured from unprocessed plant materials, i.e., grains, apples, grapes, or sugarcane (17–19). Depending on the ingredients used in their production, the fermentation processes, and the microorganisms participating in the process, different kinds of vinegar exhibit unique characteristics, flavors, and tastes (19). Numerous studies have already shown how effective the production process is concerning the final aroma characteristic of vinegar and its organoleptic properties (20, 21). Other than the production process, other factors affect the proportion of specific compounds, such as volatile and phenolic compounds, which is essential for the determination of vinegar quality (22). Recent studies on fruit vinegar production have focused on the isolation of specific acetic acid bacteria and the vinegars' phenolic and aromatic profiles to manage and improve vinegar quality (23). The ability of various bacterial strains to produce vinegar at high acetic acid concentrations has been tested (24).

Sugarcane is the world's largest source of sugar, and the main crop in many regions, and as a C_4_ plant with high energy efficiency, it is more important than other crops in terms of renewable energy utilization rate and crop production capacity. It has wide adaptability, stress tolerance, a high net energy ratio, and yield potential. Crop growth characteristics, widely planted in tropical and subtropical regions, not only as the main raw material for the sugar industry but also as a vital energy crop, the average annual biomass production is 180–200 t/ha, the yield as raw material, and ethanol production are significant due to other crops. The use of commercial promoters to initiate acetic fermentation or the implementation of cutting-edge techniques to produce high-quality sugarcane vinegar is an interesting research area discussed in this article with key importance given to the production process of sugarcane vinegar.

## History and current status of vinegar

Vinegar is used as a condiment or preservative in salad dressings, ketchups, and sauces or mixed with water for use as a beverage (22). Interestingly, vinegar was once thought to be a culinary byproduct produced when wine deteriorated from exposure to air. The first known use of vinegar was over 10,000 years ago (25, 26). French chemist Durande succeeded in creating glacial acetic acid by concentrating vinegar in the 18th century. A technique for making vinegar known as the generator process, invented by German scientist Schutzenbach in the 19th century, allowed vinegar to be produced in 7 days. German inventor Hromatka developed submerged acetification, an improved vinegar-making process that uses better aeration and stirring to develop vinegar in a shorter period (25).

In Europe, America, and Africa, fruit vinegar is usually manufactured and used as a spice (3, 5, 6, 8). Fruit vinegar is made from various fruits, including grapes, apples, pineapples, mangoes, jujubes, and bananas (16). There are many well-known traditional fruit vinegarsthroughout the world, including traditional balsamic vinegar (TBV), balsamic vinegar (BV), and sherry vinegar (SV), all of which are PGI products in Europe (27). The ancient Egyptians, Sumerians, and Babylonians are believed to have developed, prepared, and utilized fruit vinegar first, according to historical records (5, 6).

Around 1,000 BC, China produced cereal vinegar, the most widely used vinegar in China, Japan, Korea, and other Asian nations (28). Seaweed salad, sushi, boiled and steamed fish, and other dishes are frequently seasoned with cereal vinegar (3, 5, 6). Sorghum, rice, wheat, corn, barley, and other starch-rich ingredients are the primary raw materials for cereal vinegar (3, 5, 6, 8).

Due to vinegar's nutritional properties directly affecting consumers' health, more than 3.2 million liters of vinegar is consumed daily in China. The quality of vinegar has received significant attention from the Chinese government (19). As regards Chinese sugar crops, i.e., sugarcane and sugar beet, more than 85% of the perennial sugar plantation area is sugarcane, which accounts for more than 90% of the total sugar production capacity. Since 1992, Guangxi, China, has become a sugarcane cultivation and sugar production area with extensive planting and production capacity. Sugarcane planting and sucrose production account for more than 60% of the Guangxi's land use. According to the statistics of the China Sugar Industry Association, the harvest area of sugarcane in the whole region was 729,600 ha, the total sugarcane output was 49,213,200 tons, sugar production was approximately 6,287,800 tons, and total sugar production was 10.67 MMT in the country during the 2020/2021 crushing season. Guangxi has become China's leading province for sugar. It is the second-largest sugar-producing province after São Paulo, Brazil. In addition, Guangxi is also the most prominent fruit-cane-producing area in China, with an annual fruit cane planting area of more than 30,000 ha, a yield of 105–150 t/ha, and a total yearly output of over 3 million tons, most of which are sold to North (29).

The high-quality fermented product processed from sugarcane juice is more significant in promoting the sugarcane industry's development in Guangxi, China, and around the globe. The sugarcane vinegar production and quality standard system in China dramatically promotes the development of China's sugarcane processing and fermented vinegar industries and enhances the influence of sugarcane vinegar standardization. Because of the high cost of raw material processing, long duration fermentation cycle, low efficiency, and unknown product efficacy, the government of the Guangxi region developed measures to promote the secondary entrepreneurship of the sugar industry. Sugarcane raw vinegar was selected as a breakthrough point to conduct technical analysis on non-sugar-diversified products with high economic value and overcome the major bottleneck of the lack of diversified processing technologies and developments in the cane industries.

**Figure 1:**
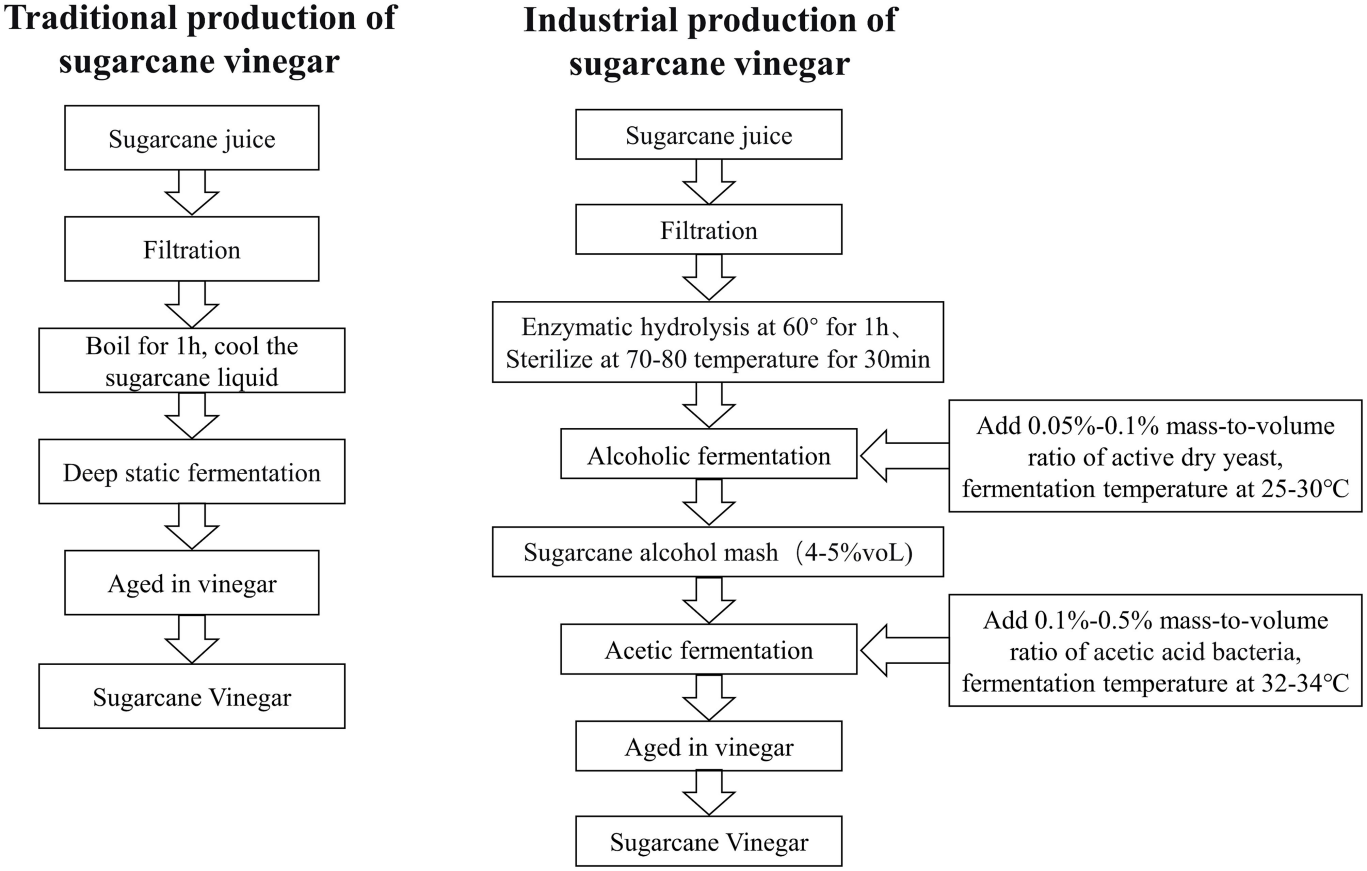
Simplified production flow diagram of sugarcane vinegar.

Sugarcane juice is a common indigenous drink, largely and economically consumed worldwide (14). Sugarcane original vinegar (SOV) developed a complete set of advanced technologies for continuous and efficient processing of original sugarcane vinegar with high efficiency of whole-stem cane juice, developed advanced equipment for different types of automatic intelligent vinegar brewing machines, and built vats for storing sugarcane brewed by immobilized microorganisms. Compared with conventional technology, the fermentation period of raw vinegar takes approximately 13–18 days, efficiency is increased up to 42.5–52.2%, and the cost is reduced by 30%, which solves the problems of the high processing cost of sugarcane raw materials and the long fermentation period. To our knowledge, this is the first time we have shown the effective retention of sugarcane original vinegar's properties and functional components during fermentation, revealing its essential biological functions (Figure 1). Different kinds of organic acids, such as amino acids, sugar, volatile components, and active ingredients of polyphenols, were assessed (Table 1). It has been confirmed that original sugarcane vinegar has the functions of lowering blood fat, improving anti-oxidative stress, reducing body weight, and enlarging organs to developing diversified new products derived from available sugarcane raw vinegar (30, 32).

**Table 1:** Summarizing aroma compounds, organic and free amino acids in sugarcane vinegar (30, 31).

**Volatile compounds**	**Relative content (%)**	**Volatile compounds**	**Relative content (%)**	**Volatile compounds**	**Relative content (%)**	**Volatile compounds**	**Relative content (%)**	**Amino acids (mg/ 100 ml)**	**Amino acids (mg/100 ml)**			**Phenolic acids**	**Alcoholic fermentation (mg/L)**	**Acetic acid fermentation (mg/L)**
Ethyl acetate	14.163 ± 0.09	3-penten-2-one	0.274 ± 0.013	Ethyl lactate	0.099 ± 0.000	Methyl benzoate	0.0385 ± 0.000	Aspartic acid (Asp)	45.10 ± 0.09	Isoleucine (Ile)	21.60 ± 0.004	Benzoic acid	1.002 ± 0.021	1.027 ± 0.070
Ethyl lactate	43.640 ± 1.263	Isoamyl alcohol	3.703 ± 0.009	N-hexanol	0.018 ± 0.000	Valeric acid	0.217 ± 0.003	Threonine (Thr)	24.30 ± 0.05	Leucine (Leu)	30.20 ± 0.004	Ferulic acid	0.205 ± 0.010	1.124 ± 0.061
Octamethylcyclotetrasiloxane	0.016 ± 0.001	Ethyl caproate	0.0785 ± 0.001	2-acetoxytetradecane	0.020 ± 0.000	Phenylethanol	0.876 ± 0.013	Serine (Ser)	19.90 ± 0.02	Tyrosine (Tyr)	11.70 ± 0.003	Quinic acid	0.074 ± 0.010	0.031 ± 0.01
Isobutyl acetate	0.068 ± 0.009	4-ethoxy-2- pentanone	0.051 ± 0.000	Nonanal	0.157 ± 0.008	N-octanoic acid	0.187 ± 0.009	Glutamic acid (Glu)	38.50 ± 0.011	Phenylalanine (Phe)	20.00 ± 0.005	Chlorogenic acid	1.635 ± 0.059	1.217 ± 0.053
Ethyl Isovalerate	0.017 ± 0.005	Octanal	0.025 ± 0.003	1,3-Di-tert-butylbenzene	0.689 ± 0.09	4-vinyl-2- methoxyphenol	0.005 ± 0.000	Proline (Pro)	9.39 ± 0.001	Lysine (Lys)	35.40 ± 0.003	Apigenin	99200.83 ± 3956	3510.88 ± 44.08
N-hexanal	0.056 ± 0.001	3-hydroxy-2- butanone	0.093 ± 0.001	Ethyl caprylate	0.413 ± 0.10	2-hydroxycinnamic acid	0.014 ± 0.000	Glycine (Gly)	29.60 ± 0.003	Histidine (His)	10.80 ± 0.006	Kaempferol	336133.64 ± 7892	3399.10 ± 104
Decane	0.049 ± 0.001	Trans-2-heptenal	0.0285 ± 0.002	Acetic acid	27.445 ± 0.401	5-hydroxymethyl furfural	0.036 ± 0.003	Alanine (Ala)	24.90 ± 0.009	Arginine (Arg)	15.60 ± 0.002	Caffeic acid	4926.56 ± 120	4715.54 ± 213
Isobutanol	0.575 ± 0.021	2-heptanol	0.013 ± 0.001	Decanal	0.118 ± 0.019	-	-	Cystine (Cys)	1.074 ± 0.001	Total amino acids (TAA)	365.77 ± 0.019	Luteolin	327692.20 ± 12384	5312.13 ± 898
Isoamyl acetate	0.496 ± 0.009	Methylheptenone	0.0065 ± 0.000	Propionic acid	0.0505 ± 0.005	-	-	Valine (Val)	25.40 ± 0.006	Essential amino acid (EAA)	159.21 ± 0.014	p-coumaric acid	15289.45 ± 1018	26600.51 ± 1159
-	-	-	-	-	-	-	-	Methionine (Met)	2.31 ± 0.002	Non-essential amino acids (NAA)	206.56 ± 0.009	-	-	-

Data are represented as mean ± SE for three (*n* = 3) biological replicates.

**Figure 2:**
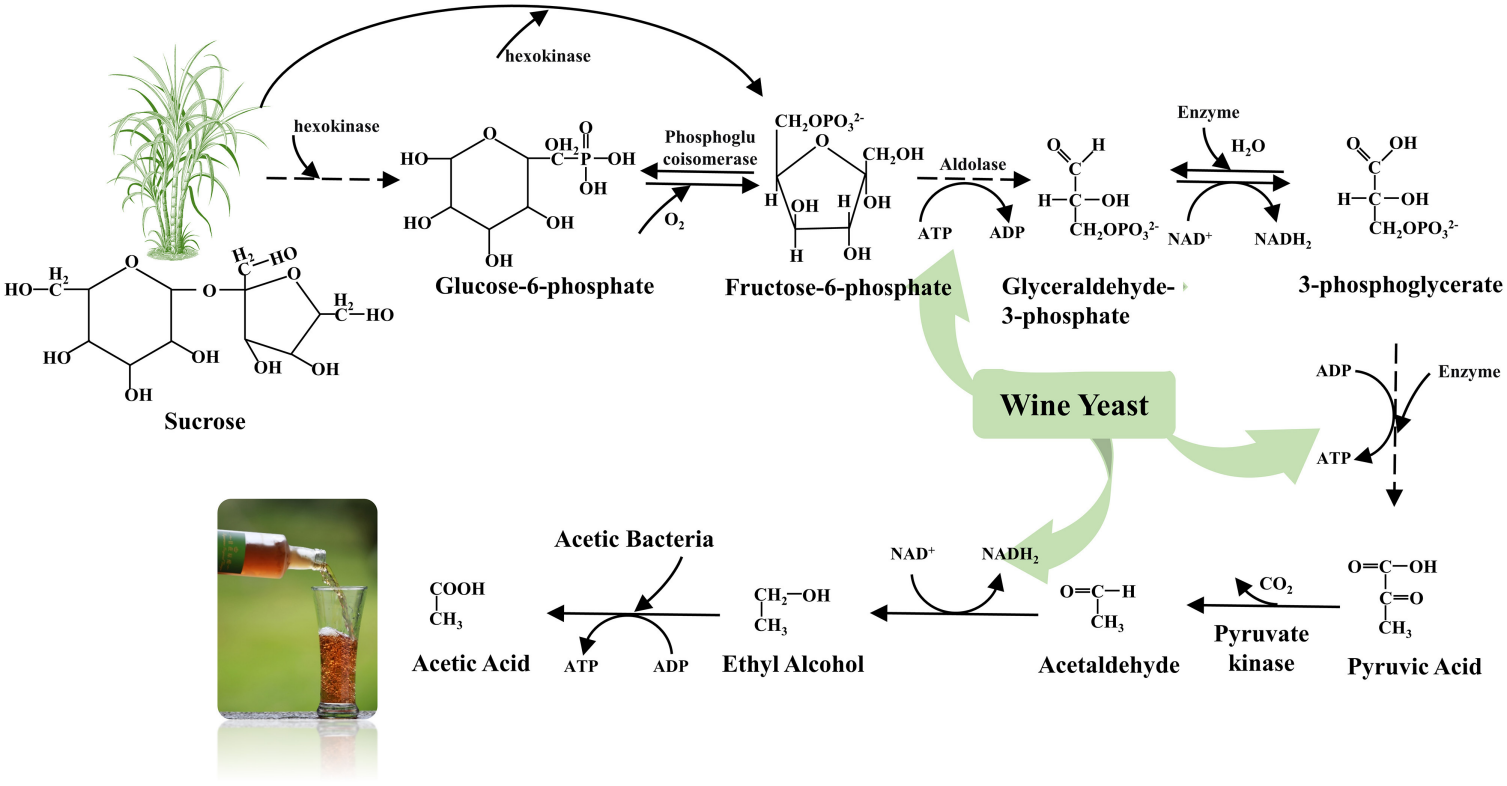
Conversion of sugarcane raw juice to acetic acid reaction.

The chemical variations in the original sugarcane vinegar produce acetic acid with two carbon atoms by the action of *Saccharomycetes* and acetic acid bacteria (catalytic enzymes) containing 12 carbon atoms of sucrose (Figure 2). The main component of raw sugarcane juice is sucrose, which is a highly suitable source of carbon for the growth of microbial activities and, thus, may be directly involved as a fermentation medium (14, 33).

## Research and development on vinegar

### Identification of yeast fermentation

The final vinegar depends on the yeast strains applied during the fermentation process. Different yeast strains can produce varying concentrations of volatile substances and alcohol. It implies that other kinds of vinegar will be produced based on the strains employed with reference to the aroma, alcohol level, and acetic acid content. The strain *S. cerevisiae* r. *bayanus* and the cider strain *S. cerevisiae* r. *cerevisiae* give rise to ciders with similar characteristics. Noteworthy in both strains is the low production of acetic acid and secondary fermentation compounds, as well as the fact that they give rise to ciders with a high concentration of glycerol and succinic acid (34). There are different methods for alcoholic fermentation, including spontaneous fermentation and inoculation with *S. cerevisiae* yeast. When compared to spontaneous fermentation, it was revealed that alcoholic fermentation with yeast could result in higher alcohol content (35). Yeast is essential in producing wines that contain higher alcohol (36).

In the analysis of wine's fermentation properties, special yeast for the alcohol fermentation of sugarcane vinegar was screened from three dry yeasts, such as wine high-activity dry yeast, high-temperature-resistant high-activity dry yeast, and highly active dry yeast for wine. According to the yeast growth curve, the delay period of three yeasts is 0–4 h, logarithmic growth period is 4–18 h, stable period is 18–28 h, which then declines after 28 h. The growth stability of yeast is determined as wine high-activity dry yeast > high-temperature-resistant high-activity dry yeast > highly active dry yeast for wine. The F coagulation value of the three yeasts was <20%, non-cohesive. Before fermentation (48 h), the CO_2_ weight loss of the three yeasts changed, indicating that the fermentation speed was fast at this stage, the yeasts were in the vigorous reproductive stage, showed strong fermentation capacity, and displayed specific difference between the fermentation time and the frequency. The acidifying ability values changed during the 3–6 days and gradually rose to a stable level during 6–8 days of fermentation. Observing the acidifying efficiency of the three yeasts in the final fermentation stage, it was determined that wine high-activity dry yeast is superior to high-temperature-resistant high-activity dry yeast (14, 37).

### Screening of acetic acid bacteria

This screening method uses sugarcane alcohol mash as raw material, through liquid submerged fermentation, LB-active acetic acid bacteria. LB-active acetic acid bacteria were selected from five acetic acid bacteria, such as bacterium and raw meal acetic acid bacteria, which are more suitable for brewing sugarcane original vinegar. The pH range between 5.5 and 6.3 is suitable for the growth of acetic acid bacteria. While some strains have been isolated from aerated media with a pH as low as 2.0, numerous investigations have demonstrated that acetic acid bacteria can still survive at pH 3.0. Three distinct strain types, known as acetophilic strains (grow at pH 3.5), acetotolerant strains (grow at pH 3.5 to 6.5), and acetophobic strains (grow at pH levels >6.5), can be explored in the production of vinegar (38). The optimal temperature for the growth of acetic acid bacteria ranged between 25 and 30°C. However, according to Raspor and Goravonic (38), acetic acid bacteria are still active at 10°C but develop more slowly.

Acetic acid is the main organic acid present in vinegar and is one of the most important functional ingredients. Acetic acid bacteria mainly produce it during fermentation. Lactic acid, which shows the highest content among nonvolatile organic acids in vinegar, is mainly produced during alcoholic fermentation. Propionic acid, tartaric acid, malic acid, citric acid, and other organic acids in vinegar are produced throughout the whole fermentation process. Moreover, the fermentation conditions also influence the contents of organic acids (7, 39). Five types of acetic acid bacteria were produced under the same situation, the acid production ability of the raw vinegar was relatively normal, and the acid production of the other four types of acetic acid bacteria showed a significant increase in the process of acetic acid fermentation (40).

### Acid production and sensory quality

Vinegar is notable for its unique aromas and flavors, mainly derived from its raw materials, microbial communities, and process technologies (3, 5, 6, 20). Because some VVOCs can emerge from one of the three sources—raw materials, microbes, and processes—or two or three of them, it is exceedingly challenging to pinpoint exactly where they originate. Additionally, VVOCs alter dynamically throughout the entire production process of vinegar. The fruit vinegar fermented by LB-active acetic acid bacteria has a sweet and sour taste, outstanding aroma, and bright color. It has the highest sensory score, which is significantly higher than those of other strains. Compared with pH, the initial alcohol content, inoculum amount, sucrose addition amount, and temperature significantly affect the acetic acid fermentation process through single-factor experiments.

### Process of alcohol fermentation

*Saccharomyces cerevisiae*, which is present in all varieties of vinegar, among the numerous microbial strains, was identified during the alcoholic fermentation process (AFP) of vinegar (3). According to Wang et al. (41), *S. cerevisiae* was the predominant yeast species in the AFP of fruit vinegar. Since *S. cerevisiae* strains grow better than other yeast species in the high-sugar environment of AFP (42). In addition to *S. cerevisiae*, LAB frequently appear in AFP and significantly contribute to the synthesis of vinegar volatile organic compounds, as do non-*Saccharomyces* like *Candida* spp., *Cryptococcus* spp., and *Debaryomyces* spp. (41, 43).

The sucrose in sugarcane raw juice was divided into glycogen for direct microaerobic fermentation. The optimal parameters were 0.1% yeast addition, 20°C fermentation temperature, 280-mL/(kg·d) oxygen flow, and 15 alcohol content of fermented mash. The sugar content of the fermented liquid with the added amount of 0.1% yeast is the highest (18.49 g/100 mL) but with a low alcohol content (14.3% vol); the apparent sugar content of the fermented liquid with the added amount of 0.2% yeast. Yeast additions of 0.15 and 0.2% bring about a faster fermentation rate. After 4 days of fermentation, the utilization rate of apparent sugar content consumption was not high and not conducive to the formation of post-fermentation sugarcane flavor. Considering the time period for fermentation comprehensively, the optimal amount of yeast was added to ferment low-alcohol sugarcane fruit wine (0.1%) (44). The amount of residual sugar in fruit wine is related to the alcohol content and the initial apparent sugar content utilization rate in the early fermentation process. Moderate residual sugar can improve the taste of fruit wine during aging and enrich the flavor of sugarcane fruit wine (14, 44).

### Acetic acid fermentation process

*Acetobacter* spp., *Komagataeibacter* spp., *Gluconobacter* spp., and *Gluconacetobacter* spp. are the dominant microorganisms in the acetic acid fermentation process of vinegar, but other bacteria, mainly *Lactobacillus* spp., *Pediococcus* spp., *Bacillus* spp., *Acinetobacter* spp., and *Staphylococcus* (8, 45, 46). These bacteria, can generate aldehydes, ketone VVOCs, and acidic VVOCs. The acidic VVOCs can be utilized as substrates to produce additional VVOCs, such as ester-like VVOCs. The primary AAB strains used to develop fruit and cereal vinegar by liquid-state and solid-state fermentation, respectively, originate from the genera *Acetobacter* and *Komagataeibacter* (5, 8).

Using sugarcane, fruit wine is prepared by fermentation of sugarcane juice as raw material through liquid-submerged fermentation. The acetic acid fermentation strains preparing sugarcane fruit vinegar from sugarcane fruit wine were screened out. The acetic acid fermentation process of sugarcane fruit wine was studied using response surface methodology (RSM). Through optimization, the optimal process parameters obtained were as follows: the initial alcohol content (5%) of sugarcane fruit wine, the inoculation amount of LB-active acetic acid bacteria (0.5%), the addition of sucrose (4%), and a fermentation temperature of 26°C. Under these conditions, the acid production of 15.05 g/100 mL was observed.

The optimum process for rapid fermentation of acetic acid bacteria was immobilized in alcoholic mash: LB-active acetic acid bacteria were used as fermentation bacteria to study the effects of initial alcohol content, inoculum size, fermentation temperature, sucrose addition, and initial pH on acetic acid fermentation and determine the most suitable LB. The initial alcohol content of active acetic acid bacteria fermentation was found to be 5% (vol), inoculum size was 0.5%, fermentation temperature was 26°C, the amount of sucrose was 4%, and initial pH was 3.6. Under these conditions, the acid production reaches 15.05 g/100 mL. The sugarcane fruit vinegar fermented by this process is amber in color, crystal clear, clear in sugarcane fragrance, and sweet and sour (40).

The interaction between initial alcohol content and inoculum volume had the most significant effect on acid production. Through regression analysis, combined with the convenience of actual operation, the processing conditions of sugarcane fruit vinegar fermented by LB-active acetic acid bacteria were determined, such as the initial alcohol content of sugarcane fruit wine was 5% vol and the inoculation amount of LB-active acetic acid bacteria was 0.5%. Compared with the predicted value of 15.57 g/100 mL, the theoretical significance and the expected value do not have a big difference, and the test results are promising, which shows that it is feasible to optimize the process conditions of fermented sugarcane fruit vinegar by response surface analysis (40).

## Organic acids and volatile compounds of vinegar

The fermentation of acetic acid is primarily responsible for vinegar's distinctive flavor and aroma. Because acetic acid is present, vinegar has a strong aroma and flavor. However, in addition to acetic acid, vinegar fermentation products, such as organic acids, esters, ketones, and aldehydes, give vinegar its unique taste (47) (Table 1). Acetic acid is a precursor for synthesizing these chemicals, produced throughout the fermentation and aging process (48). The initial raw materials utilized, the techniques for making vinegar, and the length of acetification could all impact these volatile compounds (49).

A total of 61 and 38 volatile compounds were identified in the traditional and industrial vinegar samples, according to Ozturk et al. (47), which assessed the volatile compounds present in Turkish traditional homemade vinegar and industrial vinegar. The two most volatile compounds in the conventional vinegar were α-terpineol (25%) and ethyl acetate (15%), among the other identified volatile compounds. It is interesting to note that ethyl acetate predominates in grape vinegar, but α-terpineol is undetectable from all samples of grape vinegar. Octanoic acid (15.6%) and isoamyl acetate (18.6%), or “banana odor”, were determined to be the principal volatile components in grape and pomegranate vinegar in the industrial samples (47).

According to Su and Chien (50), acetic acid (the vinegar odor), 2/3-methylbutanoic acid (sweaty odor), phenethyl acetate (sweet, honey odor), 2-phenyl ethanol (rosy, sweet odor), octanoic acid (sweaty odor), eugenol (clove odor), and phenylacetic acid were the most significant aroma-active compounds in the vinegar produced (overall floral odor). Some substances, including linalool (floral, cut grass odor), 2,3-butanedione (buttery odor), (E,Z)-2,6-nonadienal (cucumber odor), ethyl butanoate (apple, fruity odor), low concentrations or not detected by GC-MS.

A study on the volatile compounds in 56 balsamic vinegar samples, old traditional balsamic vinegar, and regular vinegar from Modena and Reggio Emilia, Italy, was conducted by Del Signore (51). Traditional balsamic vinegar contains less propionic acid and more esters than common balsamic vinegar. 2,3-Butanediol diacetate is found in higher concentrations in traditional balsamic vinegar. Conventional balsamic vinegar had higher concentrations of diacetyl, hexanal, and heptanal than balsamic, common, and other vinegar types (five times smaller in quantity). In terms of alcohol, traditional balsamic vinegar showed higher levels of octanol, whereas balsamic vinegar contained higher levels of 1-propanol, isobutyl alcohol, isoamyl alcohol, and 1-hexanol. 2-propanol and ethanol were more common in regular vinegar (51).

The maturation of the vinegar has a considerable impact on the organic acids (lactic, acetic, and succinic) and volatile substances (2-butanol, 2-propen-1-ol, 4-ethylguaiacol, and eugenol) of vinegar (17). These chemicals were discovered to be more prevalent in vinegar with increasing maturation levels. When compared to inoculated fermentation, spontaneous fermentation produces significantly higher amounts of esters during alcoholic fermentation (35).

Using HPLC-DAD technology to analyze the sugarcane original vinegar and its derivative products, sugarcane vinegar beverage is rich in phenolic substances, such as vanillin, coumarin, chlorogenic acid, caffeic acid, ferulic acid, p-coumaric acid, 10 types of luteolin in celery, cinnamic acid, and kaempferol, among which the contents of vanillin, coumarin, chlorogenic acid, and caffeic acid are relatively high (original sugarcane vinegar>5 ppm, sugarcane vinegar drink>2 ppm), the contents of apigenin, luteolin, cinnamic acid, and kaempferol are low (sugarcane original vinegar <33.99 ppm, sugarcane vinegar drink <0.1 ppm), and the total phenolic content of sugarcane vinegar was found to be higher (Table 1). The beverage types and content of polyphenols in sugarcane vinegar beverages were 2.5 times and five times higher than those of commercially available apple cider vinegar beverages and significantly higher than those of similar commercially available vinegar beverage products (52). The main components of organic acids in original sugarcane vinegar were identified by HPLC-UV, i.e., oxalic acid, tartaric acid, acetic acid, and succinic acid.

Sugarcane fruit wine and sugarcane vinegar were fermented with sugarcane mixed juice as raw materials. The automatic amino acid analyzer determined the types and content changes of amino acids in sugarcane juice, sugarcane fruit wine, and sugarcane vinegar. The nutritional and taste intensity values (TAVs) were used to compare fermented sugarcane products. It was found that sugarcane juice, sugarcane fruit wine, and sugarcane vinegar all contained different types of amino acids (such as glycine, leucine, methionine, tyrosine, histidine, threonine, alanine, isoleucine, and tryptophan acids,/lysine, aspartic acid, valine, phenylalanine, proline, serine, glutamic acid, and arginine), total amino acids (TAA), essential amino acids (EAA), and flavor amino acids, which were significantly different. Methionine and cysteine were the first limiting amino acids in sugarcane vinegar (Table 1). The ratio of EAA in sugarcane wine and sugarcane vinegar tended to be more reasonable than in sugarcane juice. Glutamic acid is the main flavor-contributing amino acid of sugarcane juice, sugarcane cider, and sugarcane vinegar, and its TAV was found to be between 1.3 and 2.4. Considering the ratio of essential amino acids, sugarcane fruit wine and sugarcane vinegar tend to be more reasonable, which can be prepared and eaten with other drinks or by developing new products to increase the biological nutritional value of the product (32).

The main sugar components (sucrose, fructose, and glucose) in original sugarcane vinegar identified by HPLC-UV make it clear that sucrose is the leading sugar source for active yeast and acetic acid bacteria to produce original sugarcane vinegar. The aroma components of original sugarcane vinegar were determined by GC-MS, such as 5 alcohols (5.19%), 9 esters (59.01%), 5 aldehydes (0.42%), and 5 acids (27.92%). Four types of ketones (0.43%), 1 type of phenol (0.01%), 3 types of hydrocarbons (0.09%), and 1 type of other heterocycle (0.69%), of which ethyl lactate, iso-acetate, isoamyl alcohol, ethyl n-caproate, ethyl octanoate, acetic acid, n-octanoic acid, etc., are the aroma substances of sugarcane original vinegar (30) (Table 1).

## Biological functions

With the help of *in vitro* and *in vivo* activity assessment, it was confirmed that the original sugarcane vinegar has biological functions, i.e., lowering blood lipid, improving anti-oxidative stress, reducing body weight, and organ enlargement. Sugarcane fruit wine and raw vinegar had a strong scavenging effect on DPPH and OH, which gradually increased with the increment of sample volume. The scavenging rate was higher than those of the control of Vc and gallic acid, among which was sugarcane raw vinegar. It may be related to the antioxidant components, such as polyphenols in the fermentation product, and the specific mechanism needs further exploration. Sugarcane fruit wine and original sugarcane vinegar have specific scavenging effects on NO_2_ and strong chelating effects on metal ions, original sugarcane vinegar is better than sugarcane fruit wine. It can be seen that original sugarcane vinegar has good health effects. Using sugarcane juice to ferment and process new products can improve sugarcane utilization value and provide an advanced way to develop diversified high-value-added products (53).

By establishing a high-fat diet-induced hyperlipidemia mouse model, the effects of sugarcane raw vinegar on blood lipids, liver lipids, and redox capacity were studied in high-fat diet-induced lipid metabolism disorders. It was found that compared with the high-fat control group, administration of original sugarcane vinegar can significantly reduce the plasma levels of total cholesterol (TC), triglyceride (TG), and low-density lipoprotein cholesterol (LDL-C) in high-fat mice, increase high-density lipoprotein (HDL-C) level, effectively reduce amylase activity, increase lipase activity, and reduce blood sugar concentration and fat accumulation; sugarcane raw vinegar can also increase the activity of superoxide dismutase (SOD) and glutathione peroxide (GSH-Px) in plasma and the liver and reduce the activity of nitric oxide synthase (NOS), and lipid peroxidation reaction products in liver malondialdehyde (MDA) content can enhance the anti-oxidative stress level (54). The differences in body weight, organ coefficients, and serum biochemical indicators between mice fed high-fat sugarcane vinegar and the model group were analyzed. It was found that the weight gain of mice fed a high-fat diet was significantly higher than that of mice in the control group. Compared with the model group, there were very significant differences. The high-concentration of sugarcane original vinegar could effectively reduce the body weight of high-fat mice; with the exception of the lung being significantly increased, other organs had no significant difference between the sugarcane original vinegar and the other groups. Sugarcane original vinegar can substantially control the body weight of mice fed a high-fat diet. A high concentration of original sugarcane vinegar can effectively reduce body weight and lower blood lipid levels and does not affect the organ index of mice. The recent experimental findings showed that original sugarcane vinegar regulates blood lipids, improves anti-oxidative stress, reduces body weight and organ enlargement, and helps to inhibit the development of hyperlipidemia, obesity, and complications (12, 31, 55).

## Social and economic benefits

Original sugarcane vinegar plays an essential role in promoting the transformation and upgradation of the cane sugar industry, stimulating the enthusiasm for sugarcane planting in China's “sea of sugarcane” and “sugar capital”, and doubling the income of sugarcane farmers. At the same time, increased employment opportunities for related industry workers in Guangxi sugarcane areas extended the sugarcane industry chain and stimulated local social and economic development. The economic and social benefits of the project are remarkable, which has promoted the development of the traditional brewing industry to standardized intelligent manufacturing and significantly promoted the upgradation of the China cane sugar industry and the process of secondary entrepreneurship around the globe.

## Conclusion and future directions

Although numerous types of volatile organic compounds (VOCs) in various kinds of vinegar, particularly in the well-known and traditional vinegar products, have been studied, the VOCs can be derived from their primary raw materials, associated microorganisms, heating, aging, or other processes. In addition, the majority of VOCs dynamically change during the vinegar-making process; therefore, it is highly challenging to explain the production processes of VOCs. Future research should at least focus on the following factors to better understand and investigate VOCs. Metagenomics, metaproteomics, and metabolomics are examples of multi-omics technologies that could be used to understand better how microbes make VOCs and how various bacteria contribute to VOC production.

## Author contributions

G-LC, F-JZ, and BL contributed to the conceptualization, methodology, investigation, resources, software, writing, and editing of the review, as well as project administration, and funding acquisition. Y-XY, X-CF, KV, and L-FY contributed to the resources, software, and data processing. All authors have read and approved the article for publication.
